# Culture of all sputum samples irrespective of quality adds value to the diagnosis of pneumococcal community-acquired pneumonia in the elderly

**DOI:** 10.1007/s10096-019-03536-9

**Published:** 2019-04-04

**Authors:** Annika Saukkoriipi, Arto A. Palmu, Jukka Jokinen

**Affiliations:** 1 Department of Public Health Solutions, National Institute for Health and Welfare, Oulu, Finland; 2 Department of Public Health Solutions, National Institute for Health and Welfare, Tampere, Finland; 3 Department of Public Health Solutions, National Institute for Health and Welfare, Helsinki, Finland

**Keywords:** Community-acquired pneumonia, Culture, Sputum, *Streptococcus pneumoniae*

## Abstract

Culture of expectorated sputum in the microbiological diagnosis of community-acquired pneumonia (CAP) is considered valid only if high-quality (HQ) samples are obtained, but evidence regarding pneumococcal etiology specifically is lacking. We studied 323 radiologically confirmed CAP cases in patients aged ≥ 65 years. Sputum samples were assessed for quality microscopically and cultured. Two quality criteria sets were applied to delineate HQ from low-quality (LQ) sputa: leukocytes/epithelial cells ratio > 5 and ≤ 2.5 epithelial cells/400× magnification field (HQ1), or leukocytes/epithelial cells ratio > 1 (HQ2). A sputum sample was obtained and the quality assessed in 224 cases; 47% were HQ1 and 76% HQ2. Encapsulated pneumococci (EPnc) were cultured in 25 (24%), 14 (12%), 35 (21%), and 4 (7%) of the HQ1-, LQ1-, HQ2-, and LQ2-samples, respectively. If another pneumococcal test (blood culture, urine antigen, or ≥ twofold increase in CbpA or PsaA antibodies) was positive, EPnc were cultured at similar proportions in HQ1- and LQ1-sputa; if the other test was negative, EPnc were cultured less often in LQ1- than HQ1-sputa. EPnc were found less often in LQ2- than in HQ2-sputa. Our results suggest similar specificity in LQ- and HQ-sputum cultures. All sputum samples add value to the pneumococcal CAP-diagnosis in the elderly.

**Electronic supplementary material:**

The online version of this article (10.1007/s10096-019-03536-9) contains supplementary material, which is available to authorized users.

## Introduction

The value of sputum culture in the microbiological diagnosis of community-acquired pneumonia (CAP) is controversial. Expectorated sputum is the most readily available specimen, but upper respiratory secretions containing oropharyngeal flora may contaminate the specimen during the collection process. Potential pathogens of the oropharyngeal flora may give a false positive result in sputum culture or bacteria of the oropharyngeal flora may overgrow pathogens of sputum resulting in a false-negative result. Therefore, culture of expectorated sputum has generally been considered worthwhile only if a sample of adequate quality, indicating that the sample is from the lower respiratory tract, has been obtained [1].

The degree of contamination of a sputum specimen by oropharyngeal flora is assessed by microscopic screening of the cellular composition of a Gram-stained smear of the specimen. Usually, the numbers of squamous epithelial cells (SECs) and polymorphonuclear cells (PMNs) are assessed [2]. If low numbers of SECs and high numbers of PMNs are found, the sample is considered to be representative of the lower respiratory tract, i.e., a high-quality (HQ) sample. A commonly used definition for adequate quality of sputum is > 25 PMNs and < 10 SECs per low power field [3–5]. However, there are variations in the criteria used and other PMNs or leukocytes to SECs ratios such as ≥ 20 [6], ≥ 10 [7], > 5 [8], ≥ 2 [9], and > 1 [10, 11] have also been applied. In a simple screen, only SECs are assessed and samples with less than a certain number of SECs per field such as 10 SECs [2, 12, 13] or 25 SECs [3] are considered as adequate. Even though there is variation in the criteria used for HQ, the sputum samples not fulfilling the criteria applied are often discarded from further analysis, which may substantially reduce the number of and bias the selection of samples analyzed by culture [4, 5, 14, 15].

To our knowledge, there are no studies evaluating the role of sputum quality regarding pneumococcal etiology specifically in the microbiological diagnosis of CAP in adults. In the present study among elderly subjects with CAP, we evaluated the relevance of sputum quality assessment for sputum culture in the diagnosis of pneumococcal CAP.

## Methods

### Study population

We studied non-institutionalized patients aged ≥ 65 years with radiologically confirmed CAP and participating in the Finnish CAP epidemiological study in 2005–2007 [16, 17]. There were altogether 323 confirmed CAP cases. We collected sputum, nasopharyngeal swab, blood for blood culture (aerobic and anaerobic) and serology (a venous blood sample with a convalescent sample 4–8 weeks later), and urine samples.

### Microbiological assessment

The processing of all samples and the methods used have been described in detail previously [16]. Regarding the sputum samples, a spontaneously produced or induced sputum sample, using a NaCl nebulizer, was obtained and processed as detailed below immediately after collection, or transferred to + 4 to + 8 °C until processing. A purulent looking part of the sputum sample was spread on a glass microscope slide and air-dried, most of the other purulent parts were homogenized with dithiothreitol (Sigma-Aldrich, St. Lous, MO), and the rest was stored for viral analyses. A part of the homogenized sputum sample was spread on a microscope slide, as above, and another part was plated undiluted and at a 1/100 dilution in 0.9% NaCl on chocolate, blood, and gentamicin blood–agar plates and incubated at 37 °C with 5% CO_2_ overnight. The glass slides and the incubated plates were sent to the bacteriology laboratory for further analysis. The glass slides were Gram-stained, and sputum quality was assessed by microscopy of the slide containing the homogenized sputum sample using a 40× objective, examining 10 fields, and calculating the average number of cells on these. The first criterion for HQ sputum sample was ≤ 2.5 SECs per field at a 400× magnification (or ≤ 10 SECs per 100× field) and a leukocytes/epithelial cells ratio > 5 (HQ1) [8, 12]. We also applied another less stringent criterion for HQ sputum sample (HQ2), according to which the sputum quality was rated high if the ratio of leukocytes/epithelial cells was > 1 [10, 11, 16]. The sputa not fulfilling the HQ1 and HQ2 criteria were considered to be of low-quality (LQ1 and LQ2, respectively). From the glass slide with unhomogenized sputum, the predominant bacterial morphologies were assessed. All sputum samples were assayed by culture regardless of the result of the quality assessment. Pneumococcus was identified from the culture plates using traditional identification methods: alpha-hemolysis, colony morphology, and optochin sensitivity (Diatabs, 10 μg, Rosco Diagnostica A/S, Taastrup, Denmark). In case of an intermediate or uncertain optochin result, a bile solubility test was used. The detection limit for sputum culture was 2 × 10^4^ colony forming units/ml of unhomogenized sputum. For serotyping, the isolates were subcultured on blood agar plates and serotyped using counterimmunoelectrophoresis, latex agglutination (neutral serogroups/types 7 and 14) and by Quellung reaction if needed using polyvalent omniserum, pool, type, group, and factor sera (Statens Serum Institut, Copenhagen, Denmark) [18]. Isolates with a positive reaction in serotyping were considered encapsulated pneumococci.

### Data analysis

Fisher’s exact test was used for the comparison of categorical variables and Mann–Whitney *U* test for continuous variables. A *p* value < 0.05 was considered statistically significant. Statistical analyses were performed using IBM SPSS Statistics for Windows, Version 25.0 (IBM Corp., Armonk, NY).

The CURB-65 score (confusion, urea > 7 mmol/l, respiratory rate ≥ 30/min, low blood pressure [systolic < 90 mmHg or diastolic ≤ 60 mmHg], age ≥ 65 years), a measure of severity of pneumonia, was calculated as described by Lim et al. [19].

## Results

A sputum sample was obtained in 226 (70%), and the quality of the sample assessed in 224 (69%) of the 323 CAP cases. In one case, the glass slide was broken, and the sputum quality could not be assessed, and in another case, there was no sample on the glass slide. The background characteristics and prognostic factors of all 323 confirmed CAP cases have been described previously [16]. The median age of the 224 cases studied here was 78 years (range 65–97 years), 54% were male, 13% were current smokers, 82% were hospitalized, and 4% died within 30 days. Of the 224 sputum samples, 106 (47%) were of HQ1 and 170 (76%) of HQ2 (Table 1). The distribution of mean numbers of leukocytes and squamous epithelial cells per field in the sputum samples are presented in Online Resource 1. Among all 323 CAP cases, an HQ1 sample was obtained in 33% of the cases and an HQ2 sample in 53% of the cases.

**Table 1:** High-quality sputum samples collected in patients with community-acquired pneumonia

Sputum samples of cases with CAP	*n*	HQ1, *n* (%)	*p*	HQ2, *n* (%)	*p*
All cases	224	106 (47)		170 (76)	
Spontaneous sputum	189	88 (47)		144 (76)	
Induced sputum	32	16 (50)	0.85^a^	23 (72)	0.66^a^
No antimicrobial exposure at the visit or in the 2 weeks prior to the visit	137	65 (47)		104 (76)	
Antimicrobial exposure at the visit	42	21 (50)	0.86^b^	33 (79)	0.84^b^
Antimicrobial exposure in the 2 weeks prior to the visit	39	18 (46)	1.00^b^	29 (74)	0.84^b^
CURB-65 score 1 to 2	168	87 (52)		131 (78)	
CURB-65 score 3 to 5	49	17 (35)	0.05^c^	33 (67)	0.14^c^

HQ1, high-quality sputum defined as a leukocytes/epithelial cells ratio > 5 and the number of epithelial cells ≤ 2.5 per field

HQ2, high-quality sputum defined as a leukocytes/epithelial cells ratio > 1

CURB-65, confusion, urea concentration of > 7 mmol/l, respiratory rate of ≥ 30/min, low blood pressure (systolic < 90 mmHg or diastolic ≤ 60 mmHg), age of ≥ 65 years [19]

^a^Comparison with the spontaneous sputum group using Fisher’s exact test

^b^Comparison with the no antimicrobial exposure group using Fisher’s exact test

^c^Comparison with the CURB score 1 to 2 group using Fisher’s exact test

Encapsulated pneumococci were cultured from 40 (18%) of the 226 sputum samples and from 25 (24%) and 14 (12%) of the 106 HQ1 and 118 LQ1 sputum samples (Fisher’s exact test for difference of proportions, *p* = 0.02), respectively, and 35 (21%) and 4 (7%) of the 170 HQ2 and 54 LQ2 sputum samples (*p* = 0.03), respectively (Table 2). Encapsulated pneumococci were cultured at similar proportions in HQ1 and LQ1 sputa, if another pneumococcal test (blood culture, urine antigen test, twofold increase in either pneumococcal surface adhesin A or choline binding protein A antibodies, encapsulated pneumococcus in nasopharyngeal swab culture) was positive (Table 2). If the other pneumococcal test was negative for pneumococcus, encapsulated pneumococci were isolated more often from HQ1 sputa than from LQ1 sputa. Encapsulated pneumococci were isolated less often from LQ2 sputa than from HQ2 sputa regardless of the results of other pneumococcal diagnostic tests.

**Table 2:** Performance of sputum culture for encapsulated pneumococcus compared to other pneumococcal diagnostic tests when using sputum samples of high quality and low quality, and of any quality

Pneumococcal test		*N*	Positive sputum culture result for encapsulated Pnc, number or proportion (%)				
			All sputum samples regardless of quality *N* = 226^a^	High-quality sputum (HQ1): leukocytes/epithelial cells ratio > 5 and the number of epithelial cells ≤ 2.5 per field		High-quality sputum (HQ2): leukocytes/epithelial cells ratio > 1	
				HQ1 sputum *N* = 106	LQ1 sputum *N* = 118	HQ2 sputum *N* = 170	LQ2 sputum *N* = 54
Sputum culture for encapsulated Pnc		226^a^	40 (18)	25/106 (24)	14/118 (12)	35/170 (21)	4/54 (7)
Blood culture for encapsulated Pnc	–	216	36 (17)^b^	24/103 (23)	11/111 (10)	32/163 (20)	3/51 (6)
	+	7	4 (57)^c^	1/2 (50)	3/5 (60)	3/5 (60)	1/2 (50)
Pnc urine antigen test	–	175	25 (14)	18/86 (21)	6/88 (7)	23/136 (17)	1/38 (3)
	+	21	8 (38)	3/7 (43)	5/14 (36)	7/14 (50)	1/7 (14)
Twofold increase in PsaA antibodies in paired sera^d^	–	158	24 (15)	18/83 (22)	6/74 (8)	23/125 (18)	1/32 (3)
	+	24	12 (50)	5/10 (50)	6/13 (46)	9/17 (53)	2/6 (33)
Twofold increase in CbpA antibodies in paired sera^d^	–	169	23 (14)	19/88 (22)	4/80 (5)	23/132 (17)	0/36 (0)
	+	23	13 (57)	5/11 (46)	7/11 (64)	10/18 (56)	2/4 (50)
Twofold increase in either PsaA or CbpA antibodies in paired sera^d^	–	162	23 (14)	18/85 (21)	5/76 (7)	22/126 (17)	1/35 (3)
	+	31	14 (45)	6/14 (43)	7/16 (44)	11/24 (46)	2/6 (33)
Positive result in at least one of the above	–	185	23 (12)	18/89 (20)	5/95 (5)	21/139 (15)	2/45 (4)
	+	41	17 (41)	7/17 (41)	9/23 (39)	14/31 (45)	2/9 (22)
NPS culture for encapsulated Pnc	–	191	16 (8)	13/89 (15)	3/101 (3)	16/144 (11)	0/46 (0)
	+	28	23 (82)	12/14 (86)	10/13 (77)	19/22 (86)	3/5 (60)

*Pnc* pneumococcus, *LQ* low quality sputum: not HQ sputum according to the criterion defined in the column, *PsaA* pneumococcal surface adhesin A, *CbpA* choline binding protein A, *NPS* nasopharyngeal swab

^a^There were 226 sputum samples that were cultured, but the sputum quality was assessed for 224 samples

^b^This refers to the false positive rate (100% − specificity), when the test is considered as the gold standard

^c^This refers to the true positive rate (sensitivity), when the test is considered as the gold standard

^d^A serum sample at the acute visit and a convalescent sample 4–8 weeks later

Thirty-two sputum samples were induced and 189 samples were spontaneously produced; in 3 cases it was not recorded whether the sputum was induced or not. The sputa were rated as HQ1 and as HQ2 at similar proportions regardless if the samples were induced or spontaneously produced (Table 1). Among the 32 induced sputum samples, encapsulated pneumococci were cultured from 5 (31%) and 2 (13%, *p* = 0.39) of the HQ1 and LQ1 samples, respectively, and from 6 (26%) and 1 (11%, *p* = 0.64) of the HQ2 and LQ2 samples, respectively. Among the 189 spontaneous sputa, encapsulated pneumococci were found in 19 (22%) and 12 (12%, *p* = 0.08) of the HQ1 and LQ1 samples, respectively, and in 28 (19%) and 3 (7%, *p* = 0.06) of the HQ2 and LQ2 samples, respectively.

In 218 CAP cases, records of antimicrobial use at the acute visit and within 2 weeks before the visit were available. Similar proportions of HQ1 and HQ2 sputa were obtained regardless if the patients had been exposed to antimicrobials prior to or at the visit or not (Table 1). Among the 137 cases with no antibiotic use, encapsulated pneumococci were isolated in 20 (31%) of the HQ1 samples and 10 (14%, *p* = 0.02) of the LQ1 samples, and in 26 (25%) of the HQ2 samples and 4 (12%, *p* = 0.15) of the LQ2 samples. There were 28 cases with antibiotic use within 2 weeks prior to the visit who were still on a course of antibiotics when enrolled in the study; encapsulated pneumococci were cultured in none of the sputum samples of these cases regardless of sputum quality.

Of the 323 CAP cases, 217 had the pneumonia severity assessed by using the CURB-65 score and a sputum sample with quality assessment available. The CURB-65 score was ≥ 1 in all cases as all patients enrolled in the present study were 65 years or older. The proportion of cases with HQ1 sputum was lower among those with a CURB-65 score of 3 to 5 (35%) than among those with a CURB-65 score of 1 to 2 (52%, *p* = 0.05, Table 1). Using the less stringent criterion HQ2 for HQ sputum, the proportions of HQ sputa among the cases with a CURB-65 score of 3 to 5 and those with a CURB-65 score of 1 to 2 were similar (Table 1).

The median age among the cases with an HQ1 sputum sample (77 years, range 65–96 years) was lower than that among the cases with an LQ1 sputum sample (80 years, 65–97 years; Mann–Whitney *U* test, *p* = 0.02). The median age among the cases with an HQ2 sputum sample (78 years, 65–97 years) and among those with LQ2 sputum (80 years, 65–97 years, *p* = 0.19) was similar.

## Discussion

This is the first study to specifically evaluate the sputum quality in the microbiological diagnosis of pneumococcal CAP in the elderly. Positive cultures for encapsulated pneumococci from HQ and LQ sputum samples using the stringent criterion showed similar concordance with other pneumococcal diagnostic tests if the other tests were positive. When the other pneumococcal tests were negative, the LQ sputum samples were more commonly negative than the HQ samples suggesting lower sensitivity rather than lower specificity. Further, the selection based on sputum quality resulted in considerable loss of cases and biased the samples towards younger and less severe cases.

The low false positive rate in LQ sputum samples observed in this study may be due to a low pneumococcal carriage rate in the study population: When carriage of encapsulated pneumococci is uncommon, contamination of sputum culture by carried encapsulated pneumococci is also unlikely. In our previous study among healthy elderly subjects (median age 70 years, range 65–92 years), the prevalence of nasopharyngeal carriage of encapsulated pneumococcus was 1.5% [20]. Other studies among the elderly have reported a prevalence of nasopharyngeal carriage of ~ 5% when conventional culture methods have been used [21, 22]. Carriage has been found to be more common, when both naso- and oropharyngeal sampling has been carried out (8%) and when molecular methods have been used (22%) [22].

The criteria commonly used to identify HQ sputum samples are based on a study published in 1975 by Murray and Washington, who showed that sputum samples with < 10 SECs per field at a 100× magnification grew the same numbers of different bacterial species as random transtracheal aspiration samples [12]. The data of Murray and Washington were later reanalyzed after removing certain species unlikely to cause pneumonia in adults, and it was concluded that sputum specimens with > 25 leukocytes per field regardless of the number of SECs were similar to transtracheal aspirates [23]. Geckler et al. [3], who studied paired sputum and transtracheal aspirate samples, found that 94% of the sputa with < 10 SECs and > 25 leukocytes, and 93% of the sputa with < 25 SECs, and containing a potential pathogen had the same organism cultured from the corresponding transtracheal aspirate. However, they also noted that if a sputum sample with < 10 SECs and > 25 leukocytes did not grow a pathogen there was still a 45% chance that the paired transtracheal aspirate would be positive for a pathogen [3]. The studies of Murray and Washington, Van Scoy, and Geckler et al. [3, 12, 23] included also other bacteria than the pneumococcus, such as enteric gram-negative rods. Previous studies have found enteric gram negative rods more often in samples taken after the initiation of antimicrobial therapy [4, 8, 14, 24], and it has been suggested that these may represent the oropharynx which may have been colonized during antibiotic treatment [8]. Thus, the criteria derived from these studies are not specifically designed for the detection of pneumococcal CAP but for detection of bacterial etiology of CAP in general. In fact, in the study of Murray and Washington, no pneumococci were recovered in the sputum specimens containing > 25 SECs and ≤ 25 leukocytes per field [12]. In a recent study among children, the prevalence of pneumococcus and other potential pathogens was compared with the quantities of SECs and PMNs in induced sputum samples [13]. It was found that the prevalence of pneumococcus decreased with increasing numbers of SECs but remained unchanged with increasing numbers of PMNs [13].

No measurable difference in the proportion of HQ samples among the induced sputa and the spontaneously produced samples was detected in the present study. This is similar to the results of a previous study, which applied a slightly different ratio of PMNs to SECs for HQ sputum but found that sputum induction did not improve specimen quality [9]. Also, sputum quality was not affected by antimicrobial treatment regardless of which one of the criteria for HQ was used. This is in concordance with the results of García-Vásquez et al. [4]. Using the same study population with CAP as in the present study, we have previously studied the effect of antimicrobial treatment on sputum culture for pneumococcus and found that antibiotics treatment given before the patients’ acute visit and still ongoing at the visit significantly reduced the detection of encapsulated pneumococci by culture in HQ2 sputum samples (only the HQ2 criterion was applied for high quality sputum in that study) [25]. In LQ2 sputa, encapsulated pneumococci were detected in none of the cases with antimicrobial treatment [25]. In the present study, regardless of sputum quality, no encapsulated pneumococci were found among the cases with prior antimicrobial treatment that was still ongoing at the acute visit.

When the more stringent HQ1 criterion was used, HQ sputum samples were less often obtained among the cases classified as more severe by using the CURB-65 score than among those with a low-severity assessment. The same was not observed when the less stringent criterion HQ2 was applied. The patients with a HQ1 sputum sample were slightly younger than the cases with a LQ1 sputum sample but there was no significant difference in the age of the patients with a HQ2 and a LQ2 sputum sample. In a previous study among adult patients using > 25 PMNs and < 10 SECs per field as the criteria for HQ, no difference in the proportions of patients with adequate sputum was observed between patients with a CURB-65 score of 1 to 2 and 3 to 5 [5]. Also, no association between the severity of CAP and the quality of the sputum sample produced was observed in previous studies in which the Pneumonia Severity Index and the Patients Outcome Research Team Score were used to assess disease severity [4, 5]. However, among elderly patients, low rates of sputum samples obtained and fulfilling the quality criteria set have been reported [14, 15]. Riquelme et al. studied 101 CAP patients aged 65 years or older and obtained a sputum sample from 47 patients but only 18 (38%) of the samples were considered valid based on their quality criteria (≥ 25 PMNs and < 10 SECs per field) [15]. Thus, only 18% of all CAP patients produced a valid sputum sample [15]. In a recent study assessing the frequency of microbiological testing of adults hospitalized with CAP on six continents, sputum culture was performed in 62% of all patients but testing practices varied significantly geographically [26]. In North and South America and Oceania, sputum culture was reported to be carried out less often and in Asia and Europe more often than in the rest of the world [26]. In the present study, a valid sputum sample according to the stringent HQ1 criterion could be obtained in 33% of all CAP cases, but in 53% when the HQ2 criterion for HQ sputum was used and in 70% when no quality criteria were used. When samples are rejected from further analyses based on prespecified criteria, the criteria applied should be carefully evaluated for the relevant pathogen(s) studied, as the rejection of samples from further analysis not only reduces the number of samples analyzed but potentially also biases the selection of cases analyzed.

In an emergency department, antimicrobial treatment is started empirically when a CAP diagnosis is likely. In the present study, the samples were collected for research purposes only and were not used in the clinical treatment of the patients. Thus, we were not able to evaluate the benefits of sputum collection regarding the patient outcomes.

In conclusion, stringent criteria for HQ sputum reduce the number of available sputum samples considerably and bias the selection of cases for microbiological analysis. However, our results suggest that the specificity of culture of LQ sputum for encapsulated pneumococcus is similar to culture of HQ sputum, which means that addition of LQ sputum culture results does not compromise the false positive rate in the diagnosis of pneumococcal CAP in the elderly. Thus, restricting the sputum samples based on arbitrary stringent quality criteria is not supported, and all samples should be evaluated for pneumococcal etiology.

## Electronic supplementary material

ESM 1 (PDF 340 kb)
