# Supplementary material for: Culture of all sputum samples irrespective of quality adds value to the diagnosis of pneumococcal community-acquired pneumonia in the elderly
Source: Eur J Clin Microbiol Infect Dis. 2019 Apr 4;38(7):1249–54. doi: 10.1007/s10096-019-03536-9 (PMC6570659; doi:10.1007/s10096-019-03536-9)
Supplement: Supplementary file 1 — (PDF 340 kb) [file 10096_2019_3536_MOESM1_ESM.pdf]

Culture of all sputum samples irrespective of quality adds value to the diagnosis of pneumococcal community-acquired pneumonia in the elderly. European Journal of Clinical Microbiology & Infectious Diseases.

By: Annika Saukkoriipi, Arto A. Palmu, Jukka Jokinen.*

**Corresponding author: National Institute for Health and Welfare, Oulu, Finland, e-mail: annika.saukkoriipi@thl.fi.*

Online resource 1

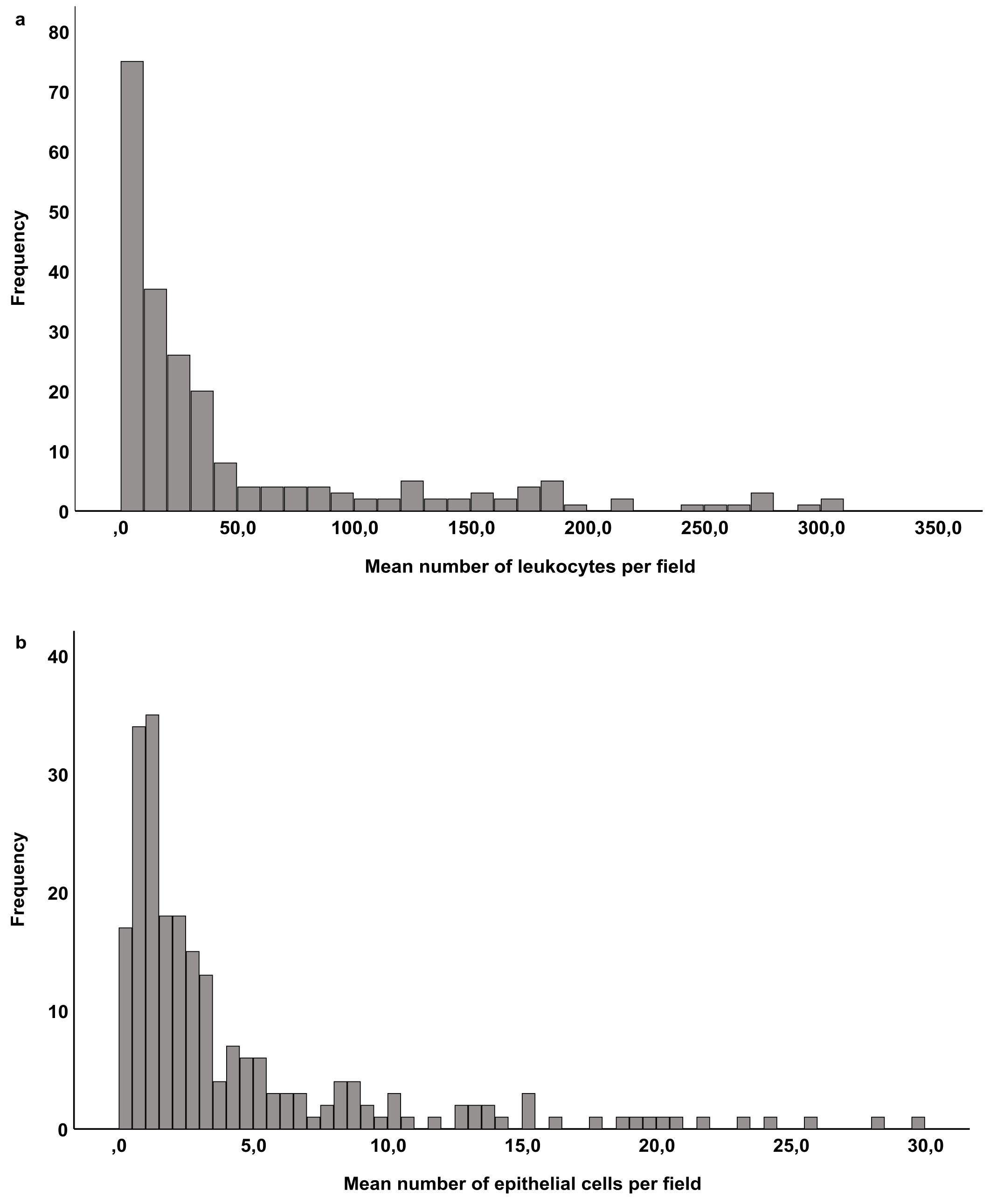

Supplementary Fig 1 The mean numbers of leukocytes per field (a) and squamous epithelial cells per field (b) in the sputum samples of the 224 cases of community-acquired pneumonia.

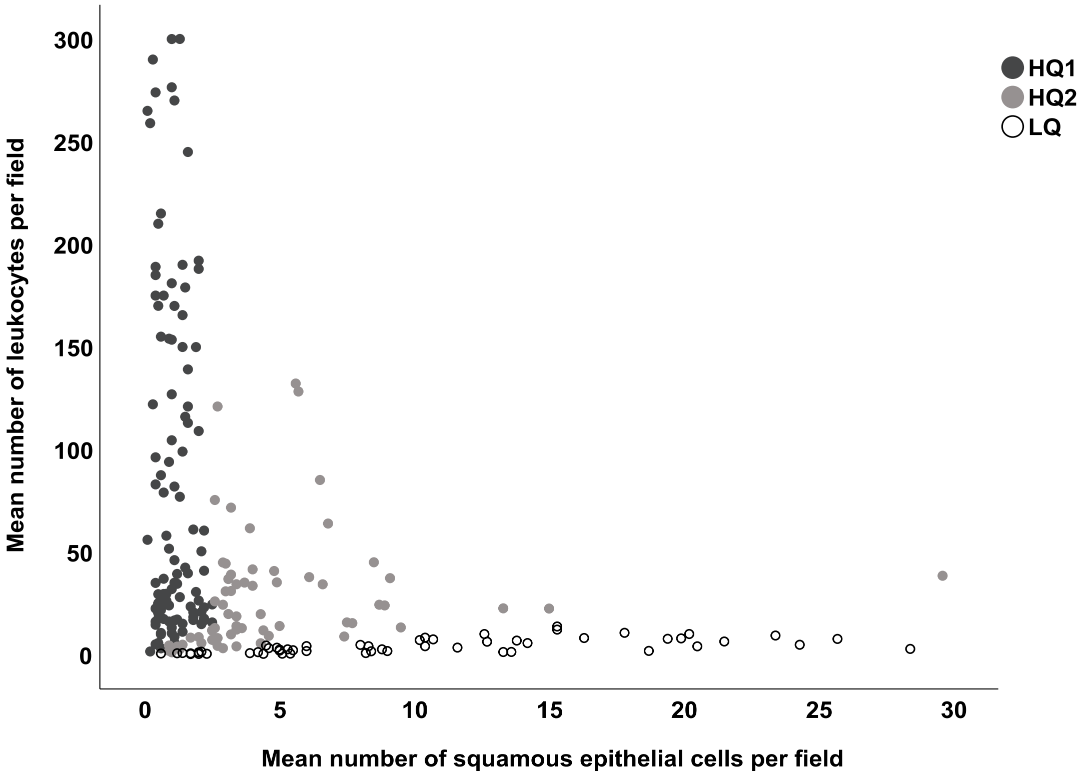

Supplementary Fig 2 The mean number of leukocytes per field versus squamous eptihelial cells per field in the 106 sputum samples fulfilling the more stringent criterion for high quality, HQ1 (≤2.5 SECs per field at a 400x magnification and a leukocytes/epithelial cells ratio >5), in the 64 sputum samples not fulfilling the HQ1 criterion but fulfilling the less stringent criterion for high quality, HQ2 (leukocytes/epithelial cells >1), and in the 54 sputum samples fulfilling neither the HQ1 criterion nor the HQ2 criterion (low-quality, LQ).
